# Supplementary material for: Effective matrix designs for COVID-19 group testing
Source: BMC Bioinformatics. 2023 Jan 24;24:26. doi: 10.1186/s12859-023-05145-y (PMC9872308; doi:10.1186/s12859-023-05145-y)
Supplement: Supplementary file 2 — Additional file 2: A detailed example. [file 12859_2023_5145_MOESM2_ESM.pdf]

A DETAILED EXAMPLE

DAVID BRUST AND JOHANNES J. BRUST

A detailed example with $N = 9,\ k = 1,\ q = p = 3,\ d = 2$

For a demonstration of how to apply our pooling test method, which consists of the construction of a pooling design and subsequent decoding, we consider a pooling design with the following parameters for simplicity: $N = 9$, $k = 1$, $q = p = 3$, $d = 2$. The detection of up to $k = 1$ positives requires $(d-1)k+1 = 2$ layers with $q^{d-1} = 3$ pools per layer. Therefore the chosen parameters lead to a pooling design capable of detecting up to one positive among nine samples denoted by $v_0 \dots v_8$ with six pools denoted by $\ell_0 \dots \ell_5$ of three samples each. The table below shows the assignment of samples into pools according to (3):

i	x	y	$qx+y$	v	ℓ
0	0	0	0	v_0	ℓ_0
1	1	0	3	v_3	ℓ_0
2	2	0	6	v_6	ℓ_0
0	0	1	1	v_1	ℓ_1
1	1	1	4	v_4	ℓ_1
2	2	1	7	v_7	ℓ_1
0	0	2	2	v_2	ℓ_2
1	1	2	5	v_5	ℓ_2
2	2	2	8	v_8	ℓ_2
0	0	0	0	v_0	ℓ_3
1	1	1	4	v_4	ℓ_3
2	2	2	8	v_8	ℓ_3
0	0	1	1	v_1	ℓ_4
1	1	2	5	v_5	ℓ_4
2	2	0	6	v_6	ℓ_4
0	0	2	2	v_2	ℓ_5
1	1	0	3	v_3	ℓ_5
2	2	1	7	v_7	ℓ_5

The assignment of samples into pools can be written as binary matrix $\mathbf{M}$ where rows correspond to pools and columns to samples:

$$\begin{array}{c} \\ \ell_0 \\ \ell_1 \\ \ell_2 \\ \ell_3 \\ \ell_4 \\ \ell_5 \end{array} \begin{array}{c} \begin{array}{ccccccccc} v_0 & v_1 & v_2 & v_3 & v_4 & v_5 & v_6 & v_7 & v_8 \end{array} \\ \left[\begin{array}{ccccccccc} 1 & & & 1 & & & 1 & & \\ & 1 & & & 1 & & & 1 & \\ & & 1 & & & 1 & & & 1 \\ 1 & & & & 1 & & & & 1 \\ & 1 & & & & 1 & 1 & & \\ & & 1 & 1 & & & & 1 & \end{array} \right] \end{array} = \mathbf{M}$$

When denoting the vectors of samples and pool test results as $\mathbf{v}$ and $\mathbf{w}$ respectively, the decoding step corresponds to the solution of the binary linear system

$$\mathbf{M}\mathbf{v} = \mathbf{w}.$$

$$\begin{aligned}
v_0 + v_3 + v_6 &= w_0\\
v_1 + v_4 + v_7 &= w_1\\
v_2 + v_5 + v_8 &= w_2\\
v_0 + v_4 + v_8 &= w_3\\
v_1 + v_5 + v_6 &= w_4\\
v_2 + v_3 + v_7 &= w_5
\end{aligned}$$

In the following demonstration we assume that sample $v_1 = 1$ is positive. After evaluation, all pools containing v_1 are positive. All samples that are included in pools that test negative, must be negative.

$$\begin{aligned}
v_0 + v_3 + v_6 &= w_0 = 0\\
v_1 + v_4 + v_7 &= w_1 = 1\\
v_2 + v_5 + v_8 &= w_2 = 0\\
v_0 + v_4 + v_8 &= w_3 = 0\\
v_1 + v_5 + v_6 &= w_4 = 1\\
v_2 + v_3 + v_7 &= w_5 = 0
\end{aligned}$$

Because pool $w_0 = 0$ tested negative, we can deduce that all samples contained therein also must be negative $v_0 = v_3 = v_6 = 0$. Analogously from pool $w_2 = 0$, we decode $v_2 = v_5 = v_8 = 0$, from pool $w_3 = 0$, we decode $v_4 = 0$ and finally from $w_5 = 0$, we decode $v_7 = 0$.

$$\begin{aligned}
v_0 + v_3 + v_6 &= w_0 = 0\\
v_1 + v_4 + v_7 &= w_1 = 1\\
v_2 + v_5 + v_8 &= w_2 = 0\\
v_0 + v_4 + v_8 &= w_3 = 0\\
v_1 + v_5 + v_6 &= w_4 = 1\\
v_2 + v_3 + v_7 &= w_5 = 0
\end{aligned}$$

Therefore we conclude that the only remaining sample must be positive $v_1 = 1$.

This example demonstrates the successful application of a pooling design capable of decoding up to $k = 1$ positives when the number of true positives does not exceed k. For this simple example the case when there are less positives than k would result in the trivial case of no positive samples leading to all negative pools.

On the other hand, when there are more positives than the design is capable of handling, it will result in false positives. At the same time, the method does not produce false negatives. When the decoding step reveals more positives than the design can identify, all positive samples need to be retested individually. This case is demonstrated in the following where we assume that samples $v_3 = v_4 = 1$ are positive. The evaluation of pools leads to the following situation:

$$\begin{aligned}
v_0 + v_3 + v_6 &= w_0 = 1\\
v_1 + v_4 + v_7 &= w_1 = 1\\
v_2 + v_5 + v_8 &= w_2 = 0\\
v_0 + v_4 + v_8 &= w_3 = 1\\
v_1 + v_5 + v_6 &= w_4 = 0\\
v_2 + v_3 + v_7 &= w_5 = 1
\end{aligned}$$

From the negative pools $w_2 = w_4 = 0$ we deduce that samples contained therein must be negative $v_1 = v_2 = v_5 = v_6 = v_8 = 0$. As no further deduction is possible at this point, the remaining four samples are marked as positive $v_0 = v_3 = v_4 = v_7 = 1$. Their number exceeds the maximum detectable number of positives $k = 1$ for this design. Therefore the four positive samples have to be retested individually.

Alternatively, a new design for larger values of k can be derived. For the detection of up to $k = 2$ positives with the other parameters of the example $q = 3$ and $d = 2$ constant, $((d-1)k+1)q = 9$ pools would be required. This is infeasible since it yields no gain with regard to the number of tests compared to testing $N = 9$ samples individually.
